# Chronic Maternal Overnutrition and Nutritional Challenge in Adult Life Disrupt Metabolic Diurnal Rhythmicity and Clock Gene Expression in Central and Peripheral Circadian Oscillators

**DOI:** 10.3390/biology14050541

**Published:** 2025-05-13

**Authors:** Lucía Carolina Cano, Erika Navarrete, Juan Pablo Ochoa-Romo, Georgina Díaz, Verónica Díaz-Hernández, Rodrigo Montúfar-Chaveznava, Ivette Caldelas

**Affiliations:** 1 Instituto de Investigaciones Biomédicas, Universidad Nacional Autónoma de México, Ciudad de México 04510, Mexico; carolina.cano@iibiomedicas.unam.mx (L.C.C.); navarrete@biomedicas.unam.mx (E.N.); ochoaromojp@iibiomedicas.unam.mx (J.P.O.-R.); ginad@iibiomedicas.unam.mx (G.D.); 2 Facultad de Medicina, Universidad Nacional Autónoma de México, Ciudad de México 04510, Mexico; roveazdih@yahoo.com.mx; 3 Facultad de Ingeniería, Universidad Nacional Autónoma de México, Ciudad de México 04510, Mexico; montufar@unam.mx

**Keywords:** maternal overnutrition, circadian rhythms, multigenerational inheritance, nutritional challenge, metabolic dysregulation, clock genes

## Abstract

In mammals, the core molecular clock genes and the overall circadian system are established during early development; during this critical period of development, maternal metabolic condition plays a major role in programming temporal metabolic regulation. Therefore, this study aimed to evaluate the effects of the chronic maternal intake of a high-fat and high-carbohydrate diet (HFCD) before and during pregnancy, in addition to a challenge with HFCD during adulthood, on offspring diurnal metabolic profile and on clock gene expression in central and peripheral circadian oscillators. The HFCD offspring and/or those exposed to the metabolic challenge exhibited alterations in the temporal profiles of analytes associated with both the carbohydrate and lipid metabolisms, as well as markers associated with liver and kidney damage, ranging from phase changes in rhythmicity or, in some cases, to the complete loss of 24 h variations. At the molecular level, the expression of clock genes (*Per1*, *Cry1*, *Bmal1*, and *Clock*) in the central and peripheral oscillators showed differential susceptibility to undergoing changes in their abundance. Our data indicate that maternal HFCD during pregnancy, a second exposure in adulthood, or both result in the long-term misalignment of the diurnal rhythm’s metabolic and damage markers; these changes are possibly associated with alterations in the core molecular circadian clockwork.

## Simple Summary

Maternal nutrition is a key factor of offspring metabolic outcome and day/night variations of these variables. In this study, we determined the effect of maternal intake of a high-fat and high-carbohydrate diet (HFCD) before and during pregnancy, in addition to subsequent exposure to an HFCD during adulthood, on offspring metabolic profile and on the expression of key genes associated with the molecular clockwork. In this study, we found that maternal poor nutrition, as well as HFCD intake during adulthood, produces alterations at the metabolic level, as well as in the molecular mechanisms that control its variations throughout the day.

## 1. Introduction

Circadian rhythmicity refers to endogenously generated 24 h fluctuations in numerous variables, from molecules to behavior. This feature, which is ubiquitous across species, endows them with the ability to anticipate the environmental day/night cycle, thus ensuring that functions occur at the optimal time. Several physiological variables, such as metabolic processes, energy balance, energy expenditure, and thermogenesis, are temporally controlled by the circadian timing system, and the continuous disruption of these variables has been associated with the development of several diseases, such as metabolic syndrome [1].

A considerable body of experimental evidence demonstrates that, in mammals, the circadian pacemaker is located in the suprachiasmatic nucleus (SCN) of the anterior hypothalamus and receives direct photic inputs from the retina that enable its photic entrainment to the alternation of the light–dark cycle [2]. In addition to generating and maintaining rhythmicity at the central level, the SCN drives peripheral oscillators that are distributed throughout the entire organism in a variety of tissues, such as the liver, heart, kidney, white adipose tissue (WAT), duodenum, and pancreas [1,3], among others. In addition, coupling between tissue-specific oscillators and the pacemaker is necessary for the optimal control of circadian physiology [4].

The mammalian circadian timing system relies on a transcriptional–translational autoregulatory feedback loop of the canonical core clock genes and their products [5,6,7]. Briefly, the activators CLOCK (circadian locomotor output cycle protein kaput) and BMAL1 (brain and muscle ARNT-like 1) heterodimerize to recognize E-box motifs to regulate the expression of different genes, such as Period (*Per1*, *Per2*, and *Per3*), Cryptochrome (*Cry1* and *Cry2*), and a set of genes known as clock-controlled genes (*CCGs*) or output genes. In the cytoplasm, the protein products PER and CRY gradually accumulate; in turn, these products are phosphorylated by casein kinases (CK1δ and CK1ε) and translocated to the nucleus, where they assemble into repressor complexes with transcriptional corepressors such as nucleosome remodeling deacetylase (*NurD*) and suppressor interacting 3A (*Sin3A*) and inhibit CLOCK/BMAL1 function [5,6,7]. The proteasomal degradation of repressor complexes leads to a decrease in PER and CRY protein concentrations, ultimately leading to the disinhibition of CLOCK/BMAL1 and restarting a new cycle [1,5,8]. In addition to the negative feedback loop, the molecular clockwork has a positive limb that includes the expression of the circadian nuclear receptor reverse erythroblastoma α (REV-ERBα; NR1D1/2) and retinoid orphan receptor α (ROR), which act as transcriptional repressors and increase BMAL1 expression [1,5,8]. In the SCN, as well as in peripheral oscillators, clock genes are abundant and rhythmically expressed.

There is reciprocal crosstalk between the circadian system and the metabolism, since critical genes associated with the control of metabolic homeostasis also participate in the core molecular circadian clock, particularly nuclear receptors such as RORα and REV-ERBα [1,5,8]. In addition, in rodents, these nuclear receptor genes are expressed in a circadian manner in key metabolic tissues that are considered peripheral oscillators, such as liver, muscle, and brown and white adipose tissue [9,10]. On the other hand, circadian core gene silencing has been implicated in the development of alterations in the temporal regulation of food intake, hyperphagia, obesity, and different symptoms associated with metabolic syndrome. In mice, the deletion of the core genes *Bmal1* or *Clock* has been associated with the development of several metabolic alterations, such as abnormal weight gain, hyperglycemia, hyperlipidemia, and hepatic steatosis [11,12,13,14]. In addition, alterations in the regulation of circadian rhythmicity, such as exposure to desynchronizing conditions such as chronic shift work or jetlag, in addition to adverse consequences on circadian timing system function, compromise homeostasis, leading to different metabolic diseases [15].

On the other hand, the intake of a high-fat and high-carbohydrate diet (HFCD), obesity, and metabolic alterations have important impacts on the generation and expression of circadian rhythmicity. Unrestricted access to a high-fat diet has important effects on circadian behaviors and the expression of neuropeptides in the hypothalamus and clock genes in the liver and adipose tissue in rodents [16]. The restriction of energy intake to certain phases of the cycle is associated with increased adiposity, glucose intolerance, and dyslipidemia [17]. Obese rats exhibit changes in their diurnal patterns of body temperature, locomotor activity, and feeding [18,19]. In experimental diabetic rodents, clock gene expression is altered in the heart, liver, and kidney, suggesting that the molecular clockwork of peripheral tissues and their mutual coupling are disrupted [20,21].

Early life (from gestation to early childhood) is considered a critical window for the establishment of molecular clock machinery and the overall circadian system. There is increasing evidence that intrauterine conditions play a major role in influencing susceptibility to certain chronic diseases in offspring. In particular, maternal overnutrition due to the chronic intake of a high-fat and high-carbohydrate diet increases the risk of the development of several noncommunicable diseases, such as hypertension, cardiovascular disease, diabetes, obesity, neurocognitive impairments, nonalcoholic fatty liver disease, and metabolic syndrome [22]. Experimental evidence obtained in murine models demonstrates that intrauterine exposure to excess nutrients can contribute to the development of obesity in offspring and to the appearance of the characteristic elements of metabolic syndrome, as well as the hepatic hyperactivation of molecular signals, which promotes the development of nonalcoholic fatty liver disease and the presence of markers of metabolic damage, such as increased phosphorylation at serine residues of IRS-1, promoting the appearance of insulin resistance [23,24].

The potential effects of chronic exposure to an HFCD during embryonic development on molecular and metabolic chronostatic regulation in adult life are poorly understood. Our research group developed a model of maternal overnutrition in rabbits since they have a lipoprotein profile and a cholesterol metabolism similar to those of humans [25], they develop similar alterations in the lipid metabolism in response to a high-fat diet [26], and they have a placental structure [27] similar to that of humans. In rabbits, maternal overnutrition causes alterations in temperature regulation in offspring, since pups obtained from mothers exposed to an HFCD exhibit a significant increase in their daily average core body temperature and alterations in their diurnal temperature rhythmicity during lactation [28]. In addition, maternal metabolic conditions before and during pregnancy have long-term effects on offspring in terms of arterial blood pressure, serum levels of analytes associated with lipid and carbohydrate metabolism, and diurnal profiles under fasting conditions in young adult rabbits (8 months old), even when the rabbits were nursed by SD-fed does and fed a standard diet after weaning. The offspring of overnourished dams exhibited considerable changes in 24 h serum metabolite profiles in adulthood, with notable sexual dimorphism [29]. The aim of this study was to determine whether the chronic maternal intake of an HFCD before and throughout the gestational period is capable of inducing long-term alterations in the temporal regulation of metabolic parameters and the expression of core clock genes in offspring.

Our research examines the hypothesis that exposure to a high-fat and high-carbohydrate diet in the pre- and/or postnatal stages affects both the metabolic set point and the diurnal profile of analytes associated with fat and carbohydrate metabolism, including the 24 h profile of damage markers of organs associated with metabolic regulation. These temporal impairments could be associated with alterations in the regulation of clock genes of central and peripheral circadian oscillators.

## 2. Materials and Methods

Our experiments were performed according to the National Institutes of Health Guide for the Care and Use of Laboratory Animals (NIH Pub. No. 86-23, revised 1996) and the research guidelines of the Instituto de Investigaciones Biomédicas, Universidad Nacional Autónoma de México (UNAM). The protocol was reviewed and approved by the Animal Care and Use Committee of the Instituto de Investigaciones Biomédicas, UNAM, before the study was conducted (ID: 198).

This study was conducted on a chinchilla strain of domestic rabbits (*Oryctolagus cuniculus*). The animals were maintained under previously described conditions [24,25,26]. Briefly, breeding rabbits (does) were housed in individual stainless-steel cages (120 × 60 × 45 cm) and maintained on a 16:8 h light–dark cycle (lights on at 09:00 h) at room temperature (20 ± 2 °C) with controlled relative humidity (40–60%).

### 2.1. Diets, Animals, and Experimental Design

The breeding rabbits and their offspring were fed a standard diet (SD; Conejo Ganador, Malta Cleyton, Mexico City, Mexico) comprising 2542.6 kcal/kg and 3.8% kcal from fat and 47.8% kcal (Table S1) from carbohydrates or an energy-rich high-fat and high-carbohydrate diet. The HFCD was composed of SD supplemented with 0.1% cholesterol (Sigma, San Luis, MO, USA), 4% soy oil (Sigma, USA), and 15% sucrose (Great Value, Mexico City, Mexico), providing 2609.2 kcal/kg and containing 5.6% kcal fat and 52.6% kcal carbohydrates (47% and 10% more energy from fat and carbohydrates, respectively, than the SD) [28,29].

**Figure 1:**
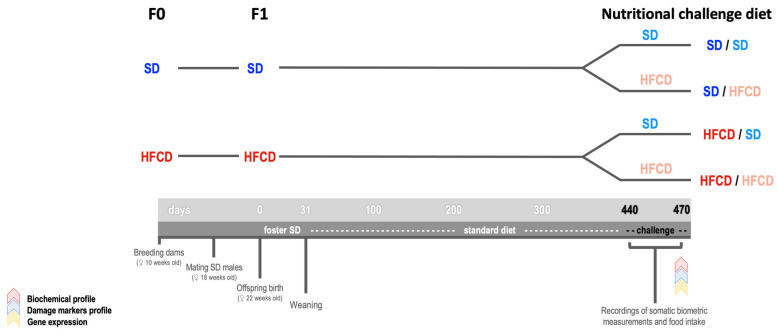
Overview of the experimental design. Nulliparous female rabbits (F0), maintained on a 16:8 h light–dark cycle, at room temperature (20 ± 2 °C), with controlled relative humidity (40–60%), were fed a standard diet (SD) or a high-fat and high-carbohydrate diet (HFCD) for eight weeks. The F0 rabbits were mated with males fed SD throughout their lives. During pregnancy, the F0 rabbits were fed the same diets; after parturition, newborn rabbits (F1) from both groups were nursed by lactating foster mothers (fed the SD) until postnatal day 31. After weaning at postnatal day 36, the F1 rabbits were fed SD and water ad libitum. At 440 days old, the F1 rabbits were challenged with an HFCD for 4 weeks, and the offspring were divided into four groups, SD/SD (*n* = 7, dark and light blue), SD/HFCD (*n* = 7, dark blue/light red), HFCD/SD (*n* = 9, dark red/light blue), and HFCD/HFCD (*n* = 10, dark red/light red). After that time, the biochemical and tissue damage markers and clock gene expression were assessed. The F1 rabbits were maintained on a 12:12 h light–dark cycle, under the same temperature and humidity conditions as those of the F0 groups.

Offspring were obtained from 10-week-old nulliparous female rabbits (*n* = 16) that were randomly assigned to one of the following groups: the F0-SD group (*n* = 8), which was fed a standard rabbit diet, or the F0-HFCD group (*n* = 8). The supplemented diet was provided for eight weeks (Figure 1). Both groups were given free access to food and water. At 140 days of age, the does were mated with 170-day-old males that were fed the previously described SD throughout their lives. During pregnancy, the SD-fed females exclusively received an SD, while the HFCD-fed does were administered both diets on alternate days to prevent miscarriages, as previously described [28,29,30].

Four days before parturition, artificial burrows were installed in each cage, and sterile hay was used to build nests. The burrows (28 × 29.5 × 30 cm high) were made of opaque polyvinyl chloride with a 14 cm diameter entrance. At birth, the rabbit pups were weighed and marked for individual identification, and the litter size was adjusted to 6 pups. The newborns obtained from both groups were exclusively nursed by lactating foster mothers (fed SD) until postnatal day 31. After weaning at postnatal day 36, all the F1 rabbits were fed an SD and water ad libitum (Figure 1). Only male F1 rabbits were used.

To determine whether the maternal diet before and during pregnancy could affect the response of the offspring to an HFCD, we challenged the F1 rabbits with an HFCD in adulthood. At 440 days of age, the offspring were divided into four groups based on their maternal diet/challenge diet: two groups of pups from SD mothers were fed either the SD or HFCD as the challenge diet, and two groups of pups from mothers fed the HFCD were fed either the SD or HFCD, resulting in the SD/SD (*n* = 7), SD/HFCD (*n* = 7), HFCD/SD (*n* = 9), and HFCD/HFCD (*n* = 10) groups. The offspring were subjected to a nutritional challenge for 4 weeks (Figure 1).

During all the nutritional challenges, the amount of food ingested during the light and dark phases was measured. For this purpose, 300 g of food was placed in the feeder every 12 h (at the beginning of the light phase and the dark phase), the remaining food was weighed at the end of each phase, and the weekly intake (kcal) was calculated. The body weights of the male rabbits in both groups were measured weekly.

After the nutritional challenge (471 days old), the rabbits were randomly sacrificed at light onset (ZT00, 09:00 h) or light offset (ZT12, 21:00 h). To obtain tissue samples, the rabbits were removed from their cages, weighed, and given an overdose of pentobarbital. The brain was quickly removed; a block containing the ventral part of the anterior hypothalamus (including the SCN) was microdissected under a stereomicroscope (Stemi 2000-C, Zeiss, Oberkochen, Germany), and a block of approximately 3 mm^3^ of tissue was obtained. Immediately after, the liver, mesenteric fat, and duodenum were removed and weighed. The liver, fat, and duodenum samples were immediately frozen in liquid nitrogen, whereas the hypothalamus was preserved in RNAlater (R901, Sigma-Aldrich, San Luis, MO, USA). All the samples were stored at −80 °C until RNA extraction.

### 2.2. Metabolic Assessment

To examine the metabolic 24 h temporal profiles of the male rabbits after the metabolic challenge (Figure 1), at 470 days of age, blood samples were obtained under fasting conditions at 09 (zeitgeber time (ZT) 0), 15 (ZT 06), 21 (ZT 12), and 03 (ZT 18) hours for the biochemical assessment of glucose (GLU), cholesterol (CHOL), low-density lipoprotein (LDL), high-density lipoprotein (HDL), free fatty acid (FFA), and triglyceride (TAG) levels. In addition, the liver damage markers of total bilirubin (T-BIL), aspartate aminotransferase (AST), alanine transaminase (ALT), and gamma glutamyl transpeptidase (GGT) were measured. The kidney damage markers urea (U), creatinine (CREA), total protein (TPRO), and albumin (A) were also measured, as was the muscle damage marker creatinine phosphokinase (CK).

Blood samples were obtained after 15 h of fasting (food was removed, but water was available). The rabbits were immobilized using a snuggle restraint, and 3 mL blood samples were harvested from the central auricular artery with a sterile 24-G catheter (Terumo, Tokio, Japan). The blood was collected in plastic serum tubes coated with silica (BD Vacutainer, Franklin Lakes, NJ, USA) and centrifuged (3000 rpm, 15 min, room temperature). The serum was stored at −70 °C until further analysis. The serum samples were processed using spectrophotometric methods, as previously described for rabbits [28,29], using commercial enzymatic colorimetric assay kits (Randox Laboratories Ltd., London, UK, and Biosino Biotechnology & Science Inc., Beijing, China). The assays were performed as recommended by the manufacturers.

### 2.3. Clock Gene Expression

To assess the expression of *Per1*, *Cry1*, *Bmal1*, and *Clock*, basal hypothalamus, liver, duodenum, and mesenteric fat tissues from male rabbits under each condition (SD/SD, SD/HFCD, HFCD/SD, and HFCD/HFCD) were collected at ZT00 and ZT12.

The tissues were homogenized using a TissueRuptor II (9002755, Qiagen, Hilden, Germany) and in TRIzol Reagent (hypothalamus-HY), RLT buffer and β-mercaptoethanol (liver-LI), or QIAzol Lysis Reagent (mesenteric fat-MF and duodenum-DU). Total RNA was isolated from the hypothalamic tissue using Direct-zol RNA MiniPrep Plus (R2072, Zymo Research, Irvine, CA, USA); for the liver analysis, an RNeasy Mini Kit Quick-Start (74104, Qiagen, Hilden, Germany) was used; for the analysis of the mesenteric fat and duodenum, an RNeasy Lipid Tissue Mini Kit (74804, Qiagen, Hilden, Germany) was used. All procedures were performed according to the manufacturer’s protocol. For all the samples, the RNA pellet was diluted in RNAse-free water, and the RNA concentration and purity were quantified using a Nanodrop ND-1000 spectrophotometer (NanoDrop Technologies, Wilmington, DE, USA). RNA integrity was examined using a 1% agarose gel run at 100 V for 40 min. 

For all tissues, complementary DNA was synthesized via reverse transcription, RNA (1 μg of HY or LI, 2 μg of MF, and 3 μg of DU) and 500 mM Exo-resistant random primer (1 μL of HY, LI, or MF, 2 μL of DU; SO181, Thermo Fisher Scientific, Waltham, MA, USA), and OligoDT (1 μL of HY, LI, or MF, 2 μL of DU; S0132, Thermo Fisher Scientific). Initially, the samples were incubated for 10 min at 65 °C, followed by a 2 min incubation on ice, after which 10 mM dNTPs were added (2 μL of HY, LI, or MF; 6 μL of DU; U1515, Promega, Madison, WI, USA), along with RT transcription buffer 5× (4 μL of HY, LI, or MF; 12 μL of DU; 3531287001, Roche, Basilea, Switzerland), reverse transcriptase (0.5 μL of HY, LI, or MF; 1.5 μL of DU; 3531287001, Roche), and RNaseOUT Recombinant RNase Inhibitor (0.5 μL of HY, LI, or MF, 1.5 μL of DU; 10000840, Invitrogen, Waltham, MA, USA) for 10 min at 25 °C, 40 min at 55 °C, and 5 min at 85 °C, respectively. The cDNA was frozen at −80 °C.

In all of the studied tissues, mRNA expression was determined via quantitative reverse transcription PCR (RT–qPCR) using a Rotor-Gene Q (Qiagen, Redwood City, CA, USA). Standard curves with serial dilutions from each tissue sample were generated via RT-qPCR assays to calculate the efficiency of the amplification reactions. For the HY, LI, DU, and MF analyses, 5 μL of cDNA (10 ng HY, 40 ng LI, 120 ng DU, and 200 ng MF), 4 μL of molecular-biology-grade water (H20MB0106, Millipore, Burlington, MA, USA), 10 μL of Taqman Master Mix 2× (4369016, Applied Biosystems, Waltham, MA, USA), and 1 μL of each Taqman assay (Taqman TM Gene Expression Assay, Thermo Scientific, Waltham, MA, USA) (Table 1) were mixed in a final volume of 20 μL. All the reactions were performed in triplicate. The thermal profile was 2 min at 50 °C, 10 min at 95 °C, 45 cycles of 15 cycles of 95 °C and 1 min at 60 °C, and 10 min at 25 °C. As endogenous controls, two genes, PPIA (Assay ID Oc03396990_g1, Thermo, Waltham, MA, USA USA) for HY, DU, and MF and H2FAV (Assay ID Oc04250259_g1, Thermo, Waltham, MA, USA USA), were used for LI. The relative expression levels of the mRNAs were calculated using the comparative 2^−∆∆CT^ method [31].

### 2.4. Data Analysis

Body weight, food and water intake, and gene expression data from the four groups were compared using one- or two-way (factors: groups and time) ANOVA, followed by the Scheffe post hoc test; the significance level considered was 5% (Statview 5.0, USA).

Data related to the differences in serum metabolite levels over time were analyzed using one-way ANOVA for independent measures (Statview 5.0, USA) to determine the potential differences associated with time, followed by a cosinor analysis (MATLAB R2023a, Carlsbad, CA, USA) to evaluate the 24 h rhythmicity of metabolic parameters [29,32]. In addition, the values of the four groups were compared using two-way ANOVA for the factors of group and time, followed by the Scheffe post hoc test (Statview 5.0, USA).

## 3. Results

### 3.1. Body Weight and Food Intake

**Figure 2:**
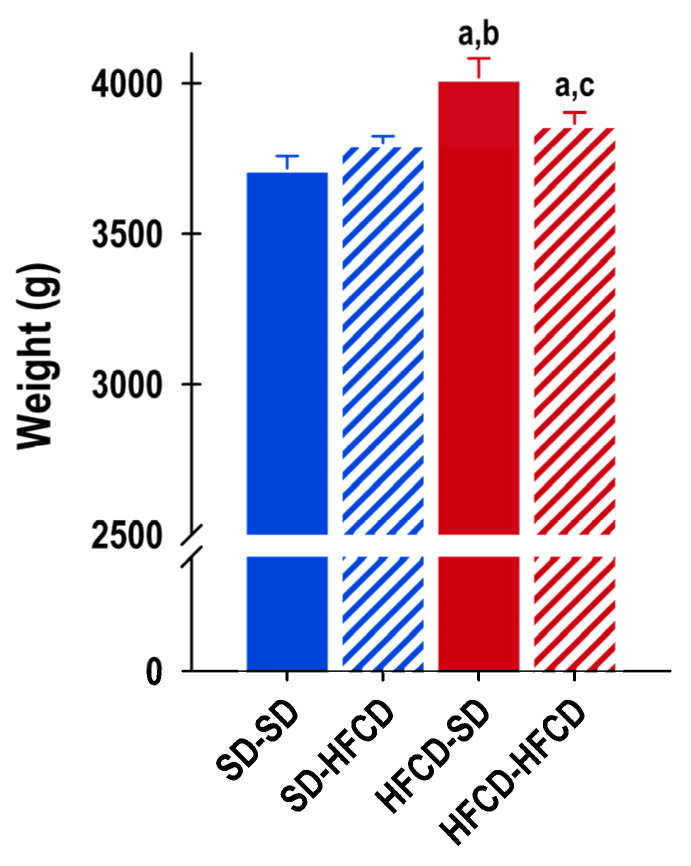
Body weight. Weights of adult rabbits obtained from mothers fed a standard diet (SD—blue bars) or a high-fat high-carbohydrate diet (HFCD—red bars) that were metabolically challenged with either the SD or the HFCD (striped blue and red bars) in adulthood. SD/SD (*n* = 7), SD/HFCD (*n* = 7), HFCD/SD (*n* = 9), and HFCD/HFCD (*n* = 10). Mean ± SEM. a denotes *p* < 0.05 vs. SD-SD, b denotes *p* < 0.05 vs. SD-HFCD, and c denotes *p* < 0.05 vs. HFCD-SD. ANOVA results (F and *p* values) of the differences associated with maternal condition and/or nutritional challenge and phase of the cycle. a denotes *p* < 0.05 vs. SD-SD, b denotes *p* < 0.05 vs. SD-HFCD, and c denotes *p* < 0.05 vs. HFCD-SD.

Maternal nutrition and metabolic challenge had significant effects on the rabbits’ body weight (F_3, 143_ = 7.5; *p* ≤ 0.0001). Compared with those from the SD-SD group, the rabbits from the overnourished mothers exhibited significant increases in body weight, and the HFCD-SD and HFCD-HFCD groups exhibited increases of 8.5% and 4%, respectively (Figure 2). The SD-SD and SD-HFCD rabbits exhibited similar trends in body weight. No changes in organ weights (normalized to animal weights) were observed in any of the groups under study.

ANOVA results (F and *p* values) of the differences associated with maternal condition and/or nutritional challenge and phase of the cycle.

**Food intake g****Food intake kcal**
**F*****p*****F*****p***Groups     0.9     NS     1.5     NS     Phase69.9<0.000163.9<0.0001Group × Time6.1<0.00016.4<0.0001

**Figure 3:**
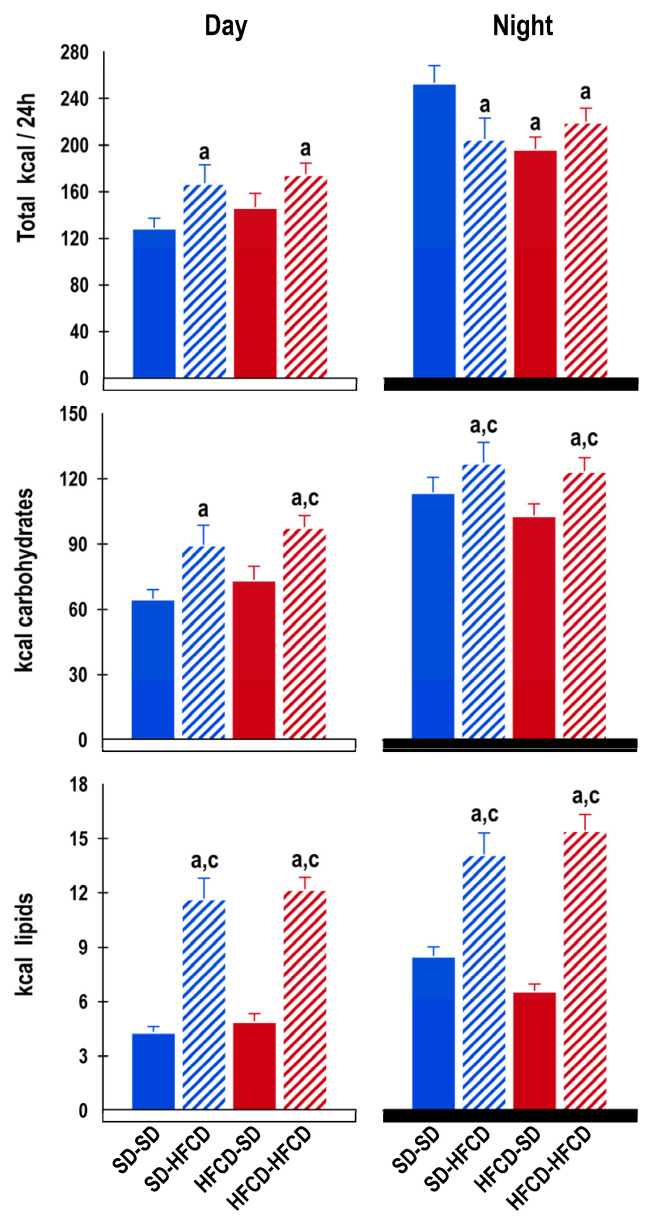
Food intake. Total kcal and average kcal contributed by carbohydrates and lipids ingested during the light and dark phases by rabbits obtained from mothers fed a standard diet (SD—blue bars) or a high-fat high-carbohydrate diet (HFCD—red bars) that were metabolically challenged with either the SD or the HFCD (striped blue and red bars). SD/SD (*n* = 7), SD/HFCD (*n* = 7), HFCD/SD (*n* = 9), and HFCD/HFCD (*n* = 10). Mean ± SEM. a denotes *p* < 0.05 vs. SD-SD, and c denotes *p* < 0.05 vs. HFCD-SD.

No significant differences in the daily amount of food (g) or total kilocalories (kcal) ingested by the rabbits were observed among the groups. During the 24 h period, the SD-SD group ate 161.1 ± 5.9 g (399.4 ± 14.7 kcal), the SD-HFCD group ate 146.4 ± 9.8 g (398.2 ± 26.3 kcal), the HFCD-SD group ate 147.6 ± 4.8 g (366.1 ± 12.0 kcal), and the HFCD-HFCD group ate 149.8 ± 4.5 (408.1 ± 12.4 kcal) per day. However, when we assessed the amount of food and kcal ingested during each phase of the cycle, significant differences were observed between groups (Figure 3). During the dark phase, the rabbits exhibited the greatest kcal observed throughout the cycle (66.4% in the SD-SD group, 55.4% in the SD-HFCD group, 57.1% in the HFCD-SD group, and 55.8% in the HFCD-HFCD group) in comparison to those in the light phase. The groups under metabolic challenge (SD-HFCD and HFCD-HFCD) showed a significant increase in the number of kcal provided by carbohydrates and lipids in comparison to the groups fed a standard diet during the light and dark phases (Figure 3).

ANOVA results (F and *p* values) of the differences associated with maternal condition and nutritional challenge.

**Dark phase****Light phase**
**F_3, 151_*****p*****F_3, 151_*****p***Total kcal     4.1     0.008     3.9     0.009     kcal/carbohydrates3.90.016.70.0002kcal/lipids42.90.00000147.20.000001

### 3.2. Temporal Metabolic Profiles

**Figure 4:**
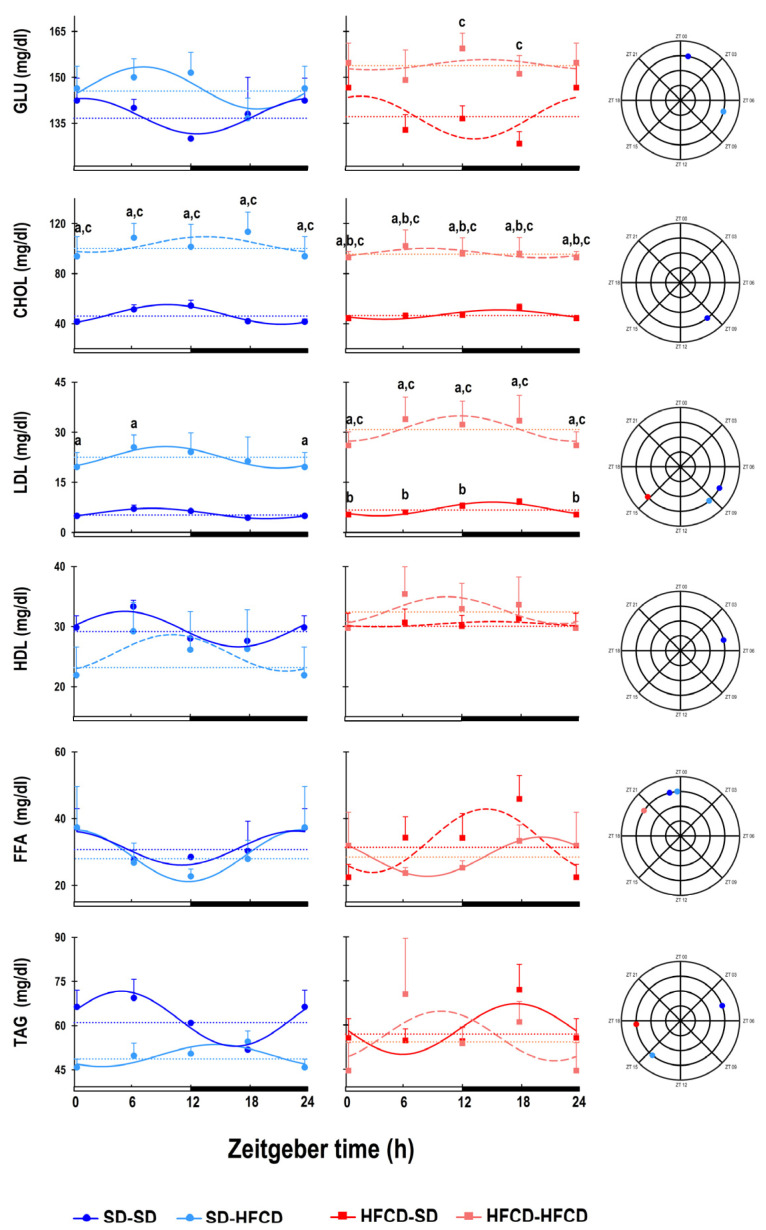
Temporal profile of metabolic patterns. Serum glucose (GLU), total cholesterol (CHOL), low-density lipoprotein (LDL), high-density lipoprotein (HDL), triglyceride (TG), and free fatty acid (FFA) levels. The curves represent the fitted cosinor function, and the mesor is indicated by the horizontal dotted line of rabbits obtained from mothers fed a standard diet (SD; dark blue dots) or a high-fat high-carbohydrate diet (HFCD; dark red squares) that were metabolically challenged with either the SD or the HFCD (light blue dots and light red squares, respectively) in adulthood. Continuous lines indicate a significant fit, and the dashed lines indicate no significant fit to a 24 h cosinusoidal function. SD/SD (*n* = 7), SD/HFCD (*n* = 7), HFCD/SD (*n* = 9), and HFCD/HFCD (*n* = 10). Mean ± SEM, a denotes *p* < 0.05 vs. SD-SD, b denotes *p* < 0.05 vs. SD-HFCD, and c denotes *p* < 0.05 vs. HFCD-SD.

Important differences associated with maternal condition and exposure to metabolic challenge were observed at the metabolic level (Figure 4). The SD-SD group exhibited significant changes in GLU levels throughout the day. According to the cosinor analysis, GLU reached its maximal level at ZT 00, and the minimum occurred at approximately ZT 12. Only exposure to the metabolic challenge during adulthood caused a significant phase delay in the daily GLU pattern at 06 h 21 m, such that, in the SD-HFCD group, the maximal plasmatic GLU levels occurred at ZT06, while the minimum plasmatic GLU levels occurred at ZT19 (Figure 4; Table S2). On the other hand, maternal overnutrition (in the HFCD-SD group) did not affect GLU levels; however, compared with the SD-SD group, maternal overnutrition caused the loss of GLU 24 h rhythmicity (Figure 4; Table S2). Among the rabbits, the most conspicuous changes in GLU patterns were observed in the group that received both gestational and adult exposures to the diet (the HFCD-HFCD group); these rabbits exhibited a significant increase of 12.2% in the mesor of GLU compared to the SD-SD group, in addition to the complete loss of 24 h rhythmicity (Figure 4; Table S2).

Regarding lipid metabolism, the CHOL levels of the SD-SD group exhibited significant changes throughout the day. According to the cosinor analysis, CHOL levels peaked at ZT 09, and the lowest CHOL levels occurred at ZT 21. In the SD-HFCD group, the metabolic challenge caused a significant increase (118%) in the level of CHOL compared with that in the SD-SD group, producing a loss of 24 h rhythmicity (Figure 4; Table S2). In the HFCD-SD group, the CHOL levels were similar to those in the SD-SD group. There was a significant increase in the mesor of CHOL in the HFCD-HFCD group (106%) compared with that in the SD-SD group, in addition to the complete loss of 24 h rhythmicity (Figure 4; Table S2).

In the LDL pattern, the SD-SD group exhibited 24 h rhythmicity, reaching its maximal level at ZT08 and its minimal level at ZT00. In the SD-HFCD group, the metabolic challenge caused a significant increase of 295% in the mesor of LDL and produced a phase delay of 01 h 26 m, so the maximal LDL levels occurred at ZT09 (Figure 4; Table S2). In the HFCD-SD group, plasmatic LDL levels were similar to those observed in the SD-SD group, but maternal overnutrition caused a significant phase delay in the LDL rhythm of 07 h 16 m, such that the maximal LDL levels occurred at ZT15, unlike those in the SD-SD group. The rabbits in the HFCD-HFCD group exhibited conspicuous changes in their LDL rhythms, characterized by a significant increase of 446% in the mesor of LDL rhythm and the complete loss of 24 h rhythmicity (Figure 4; Table S2).

In terms of the HDL rhythm, the SD-SD group exhibited 24 h rhythmicity, peaking at ZT 05 and at ZT 21. The metabolic challenge during adulthood (SD-HFCD group) caused a decrease of 13% in the serum HDL concentration and the complete loss of 24 h rhythmicity. For the HFCD-SD and HFCD-HFCD rabbits, both groups presented a complete loss of 24 h rhythmicity of HDL (Figure 4; Table S2).

In the case of FFA, the SD-SD group exhibited 24 h rhythmicity, reaching its maximal level at ZT 23 and the minimal level at ZT 11. The rhythmic pattern of the FFA in the SD-HFCD group was similar to that in the SD-SD group. Maternal overnutrition caused a complete lack of FFA 24 h rhythmicity in the HFCD-SD group. However, maternal overnutrition and metabolic challenge (HFCD-HFCD rabbits) caused a phase advance in the FFA rhythm of 03 h 44 m; therefore, the maximal FFA levels occurred at ZT 20 (Figure 4; Table S2).

ANOVA results (*p* values, * denotes *p* < 0.05) of the differences associated with nutrition (N), zeitgeber time (ZT), and the interaction between two factors (N and ZT).

**N****ZT****N • ZT**GLU0.001 *0.040.8CHOL     <0.0001 *     0.6     0.9     LDL<0.0001 *0.60.9HDL0.10.40.9TG0.50.60.7FFA0.50.50.3

For the TAG rhythmic pattern, both the maternal condition and metabolic challenge had significant effects. The SD-SD group exhibited 24 h rhythmicity in terms of TAG levels, which reached their maximal at ZT 04 and their minimal at ZT 16. For the animals exposed to metabolic challenge only during adulthood, in the SD-HFCD group, the TAG rhythm exhibited a conspicuous phase delay of 9 h 57 m. In contrast, the rabbits in the HFCD-SD group exhibited a conspicuous phase advance of the TG pattern of 10 h 58 m, and the acrophase occurred at ZT 17. The rabbits in the HFCD-HFCD group displayed a complete loss of 24 h TAG rhythmicity (Figure 4; Table S2).

### 3.3. Temporal Profile of Tissue Damage Markers

**Figure 5:**
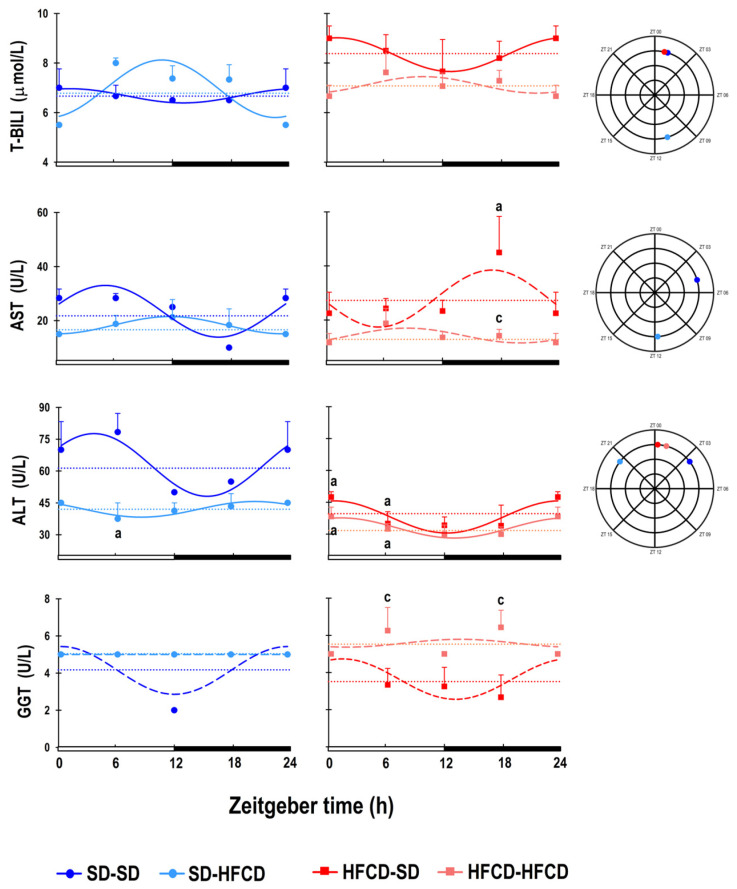
Temporal profile of liver damage markers. Plasmatic levels of total bilirubin (BILI-T), aspartate aminotransferase (AST), alanine aminotransferase (ALT), and gamma-glutamyl transferase (GGT). The curves represent the fitted cosinor function, and the mesor is indicated by the horizontal dotted line of rabbits obtained from mothers fed a standard diet (SD; dark blue dots) or a high-fat carbohydrate diet (HFCD; dark red squares) that were metabolically challenged with either the SD or the HFCD (light blue dots and light red squares, respectively) in adulthood. SD/SD (*n* = 7), SD/HFCD (*n* = 7), HFCD/SD (*n* = 9), and HFCD/HFCD (*n* = 10). Continuous lines indicate a significant fit, and the dashed lines indicate no significant fit to a 24 h cosinusoidal function. Mean ± SEM, a denotes *p* < 0.05 vs. SD-SD, and c denotes *p* < 0.05 vs. HFCD-SD.

According to the analysis of the liver damage markers, the SD-SD group exhibited significant changes in T-BILI levels throughout the day, peaking at ZT 01 and reaching their minimum at ZT 13. In the SD-HFCD group, the metabolic challenge caused a prominent change in rhythmicity, such that the T-BILI rhythm was in an antiphase, with maximal levels occurring at ZT 10. In the HFCD-SD group, the 24 h pattern showed similar results to those observed in the SD-SD group (an acrophase at ZT 01 and the nadir at ZT 12), except for the mesor, which was 23% greater than that observed in the SD-SD group. The HFCD-HFCD rabbits exhibited a complete loss of 24 h T-BILI rhythmicity (Figure 5; Table S3).

Regarding AST plasmatic levels, the SD-SD group exhibited 24 h rhythmicity, with maximal levels occurring at ZT 04 and minimum levels at ZT 17. The mesor of AST in the SD-HFCD group was similar to that in the SD-SD group, but the rhythm exhibited a phase delay of 6 h 53 m (Figure 5; Table S3). For the HFCD-SD and HFCD-HFCD rabbits, maternal overnutrition and/or metabolic challenge caused a lack of 24 h rhythmicity in AST plasmatic levels; in the mesor, the HFCD-SD group exhibited an increase of 19.2% in AST levels; conversely, the HFCD-HFCD group showed a decrease of 38% in AST levels (Figure 5; Table S3).

For ALT levels, the SD-SD group exhibited 24 h rhythmicity, with maximal ALT levels occurring at ZT 03 and minimal levels at ZT 15. The metabolic challenge caused alterations in the rhythm, since the SD-HFCD group exhibited a phase advance of 06 h 58 m of the ALT diurnal pattern. The 24 h pattern of ALT was similar in the HFCD-SD and HFCD-HFCD rabbits, with their maximal plasmatic levels occurring at the beginning of the light phase (ZT 00 and 01, respectively) and their nadir at the beginning of the dark phase (ZT 12); thus, both groups exhibited a phase advance of the rhythm (3 h 14 m and 0 h 28 m, respectively) in comparison to the SD-SD group. The ALT levels in the SD-HFCD, HFCD-SD, and HFCD-HFCD groups were significantly lower (approximately 33%, 39%, and 47%, respectively) than those in the SD-SD group (Figure 5; Table S3).

Neither of the groups under study showed 24 h rhythmicity in GGT plasmatic levels. Compared with those in the SD-SD group, GGT levels in the HFCD-SD group decreased by approximately 12%, whereas GGT levels in the SD-HFCD and HFCD-HFCD groups increased by approximately 21% and 36%, respectively (Figure 5; Table S3).

ANOVA results (*p* values, * denotes *p* < 0.05) of the differences associated with nutrition (N), zeitgeber time (ZT), and the interaction between two factors (N and ZT).

**N****ZT****N • ZT**BILI T     0.06     0.8     0.9     AST0.007 *0.90.3ALT<0.001 *0.20.7GGT0.01 *0.40.7

**Figure 6:**
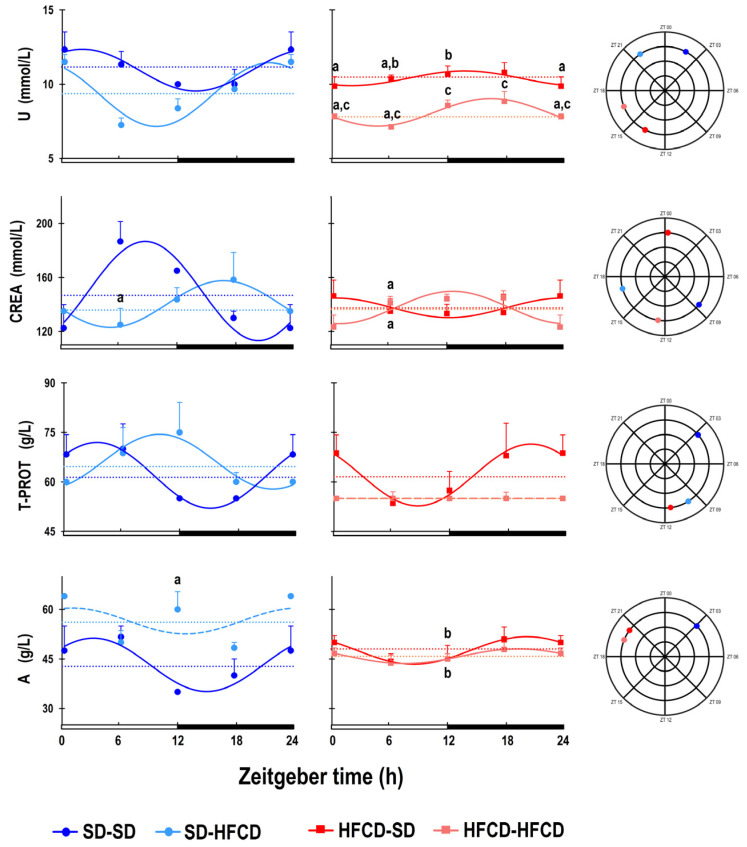
Temporal profile of renal damage markers. Urea (U), creatinine (CREA), total protein (T-PROT), and albumin (A) plasmatic levels. The curves represent the fitted cosinor function, and the mesor is indicated by the horizontal dotted line of rabbits obtained from mothers fed a standard diet (SD; dark blue dots) or a high-fat carbohydrate diet (HFCD; dark red squares) that were metabolically challenged with either the SD or HFCD (light blue dots and light red squares, respectively) in adulthood. SD/SD (*n* = 7), SD/HFCD (*n* = 7), HFCD/SD (*n* = 9), and HFCD/HFCD (*n* = 10). Continuous lines indicate a significant fit, and the dashed lines indicate no significant fit to a 24 h cosinusoidal function. Mean ± SEM, a denotes *p* < 0.05 vs. SD-SD, b denotes *p* < 0.05 vs. SD-HFCD, and c denotes *p* < 0.05 vs. HFCD-SD.

In terms of renal damage markers, according to the cosinor analysis of the SD-SD group, the plasmatic levels of U exhibited 24 h rhythmicity, with maximal levels occurring at ZT 01 and nadir levels occurring at ZT 14. Among the rabbits that were exposed to metabolic challenge, the SD-HFCD group exhibited a phase advance of 4 h 10 m in the U rhythm, with maximal levels occurring at ZT 21 and lower levels occurring at ZT 10. The 24 h patterns of U were similar for the HFCD-SD and HFCD-HFCD rabbits, with the maximal U concentration occurring at the beginning of the dark phase (ZT 13 and 16, respectively) and the nadir occurring at the beginning of the light phase (ZT 02 and 04, respectively); thus, both groups exhibited a phase delay in the rhythm (11 h 54 m and 13 h 32 m, respectively) compared with the SD-SD group (Figure 6; Table S4). The U levels in the SD-HFCD, HFCD-SD, and HFCD-HFCD groups were significantly lower (approximately 14%, 4%, and 25%, respectively) than those in the SD-SD group (Figure 6; Table S4).

ANOVA results (*p* values, * denotes *p* < 0.05) of the differences associated with nutrition (N), zeitgeber time (ZT), and the interaction between two factors (N and ZT).

**N****ZT****N • ZT**U     <0.001 *     0.04 *     0.02 *     CREA0.40.30.02T PROT0.80.80.2A0.003 *0.20.06

The plasmatic levels of CREA showed 24 h rhythmicity in SD-SD rabbits, with maximal levels occurring at ZT08 and the nadir close to ZT20. The SD-HFCD and HFCD-HFCD groups exhibited a phase delay in the CREA rhythm of 8 h 21 m and 03 h 58 m, respectively, whereas the HFCD-SD group exhibited a phase advance of 8 h 22 m in comparison to the SD-SD group (Figure 6; Table S4).

According to the results of the cosinor analysis, the serum T-PROT exhibited 24 h rhythmicity in the SD-SD group, and the maximal levels were recorded at ZT 03, while the nadir was recorded at ZT 15. No significant changes in the mesor were observed in the SD-HFCD rabbits that were exposed to the metabolic challenge, but, in the 24 h pattern, T-PROT exhibited a phase delay of 6 h at 37 m, with the maximal levels occurring at ZT09 (Figure 6; Table S4). Maternal overnutrition affects the T-PROT rhythm, since the HFCD-SD group exhibited a phase delay in the rhythm of 17 h 22 m compared with that of the SD-SD group. The HFCD-HFCD group lacked 24 h rhythmicity in terms of the serum T-PROT (Figure 6; Table S4).

The plasmatic levels of A exhibited 24 h rhythmicity in SD-SD rabbits, with maximal levels occurring at ZT 03 and the nadir occurring at ZT 15. The exposure to the metabolic challenge in the SD-HFCD group caused a significant increase of 31% in the mesor of the rhythm and a lack of 24 h rhythmicity in plasmatic A levels. The HFCD-SD and HFCD-HFCD groups exhibited similar 24 h rhythms, with a discrete increase in mesor (10% and 6%, respectively) and a phase delay in the A rhythm of 17 h 21 m and 16 h 24 m, respectively (Figure 6; Table S4).

**Figure 7:**
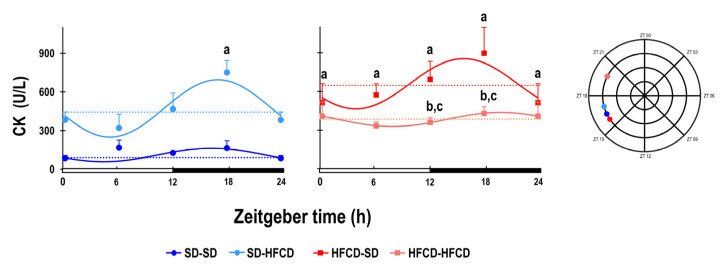
Temporal profile of muscle damage markers. Plasmatic levels of creatinine kinase (CK). The curves represent the fitted cosinor function, and the mesor is indicated by the horizontal dotted line of rabbits obtained from mothers fed a standard diet (SD; dark blue dots) or a high-fat high-carbohydrate diet (HFCD; dark red squares) that were metabolically challenged with either the SD or HFCD (light blue dots and light red squares, respectively) in adulthood. SD/SD (*n* = 7), SD/HFCD (*n* = 7), HFCD/SD (*n* = 9), and HFCD/HFCD (*n* = 10). Continuous lines indicate a significant fit, and the dashed lines indicate no significant fit to a 24 h cosinusoidal function. Mean ± SEM, a denotes *p* < 0.05 vs. SD-SD, b denotes *p* < 0.05 vs. SD-HFCD, and c denotes *p* < 0.05 vs. HFCD-SD.

Finally, for the muscle damage marker creatinine kinase, according to the cosinor analysis of the SD-SD group, the plasmatic levels of CK exhibited 24 h rhythmicity, with maximal levels occurring at ZT 16 and the nadir at ZT 03 (Figure 7; Table S5). Among the rabbits that were exposed to metabolic challenge during adulthood, the SD-HFCD group exhibited a significant increase of 326% in the mesor of CK, with a discrete phase delay of 42 m in the rhythm, with maximal levels occurring at ZT 16 and the nadir occurring at ZT 05. The CK levels were significantly greater in the HFCD-SD and HFCD-HFCD groups (496% and 245%, respectively) than in the SD-SD group. The HFCD-SD group showed a phase advance of 33 m in the CK rhythm, while the HFCD-HFCD group had a phase delay of 3 h 32 m in the rhythm (Figure 7; Table S5).

ANOVA results (*p* values, * denotes *p* < 0.05) of the differences associated with nutrition (N), zeitgeber time (ZT), and the interaction between two factors (N and ZT).
     **N**     **ZT**     **N • ZT**     CK<0.001 *0.05 *0.8

### 3.4. Central and Peripheral Clock Gene Expression

**Figure 8:**
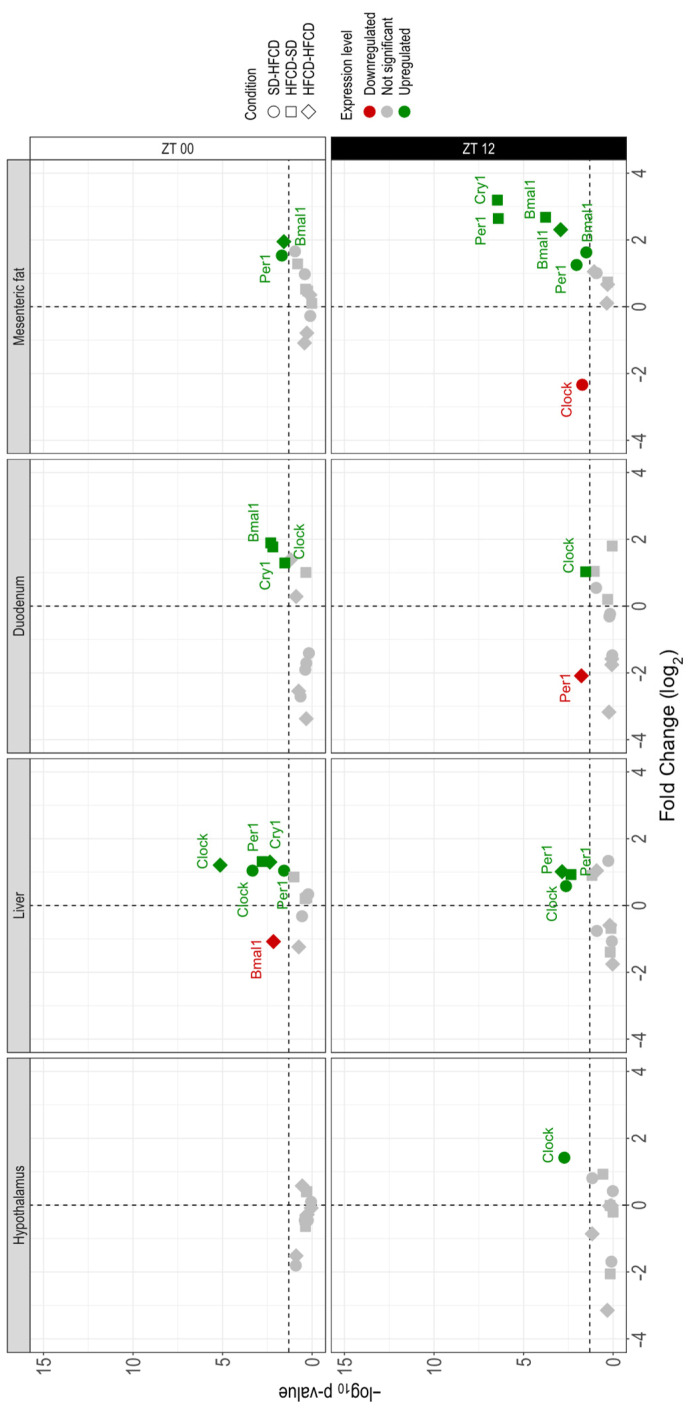
Clock gene expression. Volcano plot shows fold change expression of the *Per1*, *Cry1*, *Bmal1*, and *Clock* genes in the hypothalamus, liver, duodenum, and mesenteric fat of adult rabbits obtained from mothers fed a standard diet or a high-fat high-carbohydrate diet (HFCD; circles) that were metabolically challenged with either the SD (squares) or HFCD (diamonds) as the challenge diet in adulthood. The X-axis represents the fold change in expression of each gene, where zero represents no change, positive numbers indicate upregulated genes (green), and negative numbers indicate downregulated genes (red); genes that are not statistically significant are represented in gray. Y-axis shows the -log10 *p* value; above the horizontal dashed line (*p* < 0.05), nonsignificant expressions are shown below the horizontal dashed line (*p* ≥ 0.05). The data were obtained in triplicate, and the mean values are shown. SD/SD (*n* = 7), SD/HFCD (*n* = 7), HFCD/SD (*n* = 9), and HFCD/HFCD (*n* = 10). The differentially expressed genes from each group are shown above the horizontal dashed line (*p* < 0.05), and the nonsignificant expressed genes are shown below the horizontal dashed line (*p* ≥ 0.05).

In the basal hypothalamus of the SD-SD group, the abundance of *Per1* was significantly greater at the beginning of the light phase than during the dark phase. However, in this group, the gene expression of *Cry1*, *Bmal1*, and *Clock* was similar at the two time points of the cycle (Figure S1). In the hypothalamus of the SD-HFCD, the expression of Clock was significantly upregulated during the dark phase in comparison to the SD-SD group. For the rest of the groups (HFCD-SD and HFCD-HFCD) none of the genes under study exhibited changes according to time or in comparison to the control condition (Figure 8).

In the livers of the SD-SD group, at the beginning of the light phase, the expression of *Bmal1* was significantly greater than at the beginning of the dark phase; on the other hand, at this time, the expression levels of the *Cry1* and *Clock* genes were greater in the livers. In contrast, no changes in *Per1* expression were detected in the livers of the SD-SD rabbits (Figure S1). In the livers of the SD-HFCD group, the expression of *Per1* was significantly upregulated during the light phase. In contrast, for *Cry1* and *Bmal1*, no changes in comparison to the control condition were detected (Figure 8). Maternal conditions had significant effects on liver *Per1* expression in the HFCD-SD group, since *Per1* was upregulated at both time points in comparison to the SD-SD group. No changes associated with the expression of the remaining clock genes were observed (Figure 8). In the HFCD-HFCD group, differential effects on gene expression in the liver were observed according to the time of day. At the beginning of the light phase, *Cry1* and *Clock* expression levels were upregulated, and *Bmal1* expression was downregulated, whereas, at the beginning of the dark phase, only *Per1* expression was upregulated in the liver compared with the SD-SD group (Figure 8).

In the duodenum of the SD-SD group, *Clock* gene expression was significantly higher at the beginning of the light phase. No changes associated with the phase of the cycle were observed for *Bmal1* and *Cry1* expression in the duodenum of the SD-SD rabbits (Figure S1). Exposure in adulthood for 30 days had no significant effect on clock gene expression in the duodenum of the SD-HFCD group compared with that of the control group (Figure 8). In contrast, in the group of malnourished dams (the HFCD-SD group), the expression of the *Cry1*, *Bmal1*, and clock genes was significantly upregulated at the beginning of the light phase; meanwhile, at the beginning of the dark phase, *Clock* expression remained upregulated in comparison to that in the control group (Figure 8). No significant changes in *Per1* expression were detected in the duodenum of the HFCD-SD group. For the group exposed to maternal overnutrition and nutritional challenge, the only significant changes in the duodenum were observed at the onset of the dark phase, with a downregulation in the expression of *Per1* in comparison to that in the control group (Figure 8). No significant changes were observed in the expression of the remaining clock genes in the duodenum of the HFCD-HFCD group.

ANOVA results (*p* values, * denotes *p* < 0.05) of the differences associated with nutrition (N), zeitgeber time (ZT), and the interaction between two factors (N and ZT).
Hypothalamus
**N****ZT****N • ZT***Per1*     
0.2     0.05 *     0.4     *Cry1*0.30.01 *0.2*Bmal1*0.20.10.1*Clock*0.20.30.006 *Liver
**N****ZT****N • ZT***Per1*0.001 *0.1<0.001 **Cry1*0.20.01 *0.002 **Bmal1*0.04<0.001 *0.1*Clock*<0.001 *<0.001 *0.005 *Duodenum
**N****ZT****N • ZT***Per1*0.02 *0.05 *0.5*Cry1*0.003 *0.04 *0.1*Bmal1*0.01 *0.005 *0.1*Clock*<0.001 *0.70.6Mesenteric fat
**N****ZT****N • ZT***Per1*<0.001 *0.004 *<0.001 **Cry1*<0.001 *0.04 *<0.001 **Bmal1*<0.001 *0.04 *0.01 **Clock*<0.001 *<0.001 *<0.001 *

In the mesenteric fat of the SD-SD group, clock gene expression was similar in both phases of the cycle associated with time (Figure S1). In the SD-HFCD group, significant changes in gene expression were observed according to the phase of the cycle; in the dark phase, *Bmal1* expression was upregulated compared to the SD-SD group (Figure 8). In the HFCD-SD group, the main effects were observed at the beginning of the dark phase, with significant upregulation of *Cry1* and *Bmal1* expression in mesenteric fat. At the beginning of the light phase, no significant changes were observed in the expression of the clock genes under study (Figure 8). Finally, the expression of *Bmal1* in the fat of the HFCD-HFCD group was significantly upregulated at both time points of the day in comparison to that observed in the SD-SD group (Figure 8). No significant changes were observed in the expression of the remaining clock genes.

## 4. Discussion

In our current study, the HFCD offspring and offspring exposed to the metabolic challenge presented alterations in the temporal profiles of analytes associated with both the carbohydrate and lipid metabolism, as well as markers associated with liver and kidney damage, including phase changes in rhythmicity or, in some cases, to the complete loss of 24 h variations. At the molecular level, the expression of clock genes in the central and peripheral oscillators showed differential susceptibilities to alterations according to the moment that the exposure to the HFCD occurred.

None of the groups that were exposed to the HFCD developed an obese phenotype; nevertheless, at the metabolic level, they exhibited dyslipidemia, hyperglycemia, or changes in markers of tissue damage. These changes were dependent on the moment that the rabbits were exposed to the HFCD. These changes are not attributable to differences in body weight, as has been previously reported in murine models in which rat offspring that were obtained from obese dams and/or exposed to high-fat diets postnatally exhibited metabolic impairments prior to alterations in body composition, such as increases in body weight or adiposity [33,34,35,36,37,38]. These findings indicate that, in mammals such as lagomorphs and rodents, an obese phenotype is not a mandatory prerequisite for the development of metabolic syndrome.

In mammals, energy intake is under circadian regulation, such that food and water intake occur predominantly during the waking period; the timing of feeding patterns is known to be critical for metabolic homeostasis [39]. In the case of rabbits, food intake increases a couple of hours prior to the onset of the dark phase and remains elevated throughout the dark phase [40]. However, in our study, there were evident changes in the diurnal pattern of food intake in the groups that were exposed to the metabolic challenge in adulthood, since, during the light phase and during their rest phase, the SD-HFCD and HFCD-HFCD groups exhibited a conspicuous increase in the intake of kcal provided by carbohydrates and lipids, which is indicative of a short fasting period and a long feeding interval. Similar findings have been reported in relation to the feeding pattern of rodents exposed to high-fat diets pre- and/or postnatally, since, in this nocturnal species, a high-fat diet produced a conspicuous increase in daytime feeding [38,41]. In rodents, it has been widely demonstrated that high-fat diets alter feeding rhythms through perturbations in cellular metabolic processes and pathways controlled by the circadian system, which, in combination with the quality of nutrients, predispose individuals to metabolic diseases [42].

As in other mammals, in rabbits, the plasmatic levels of analytes associated with the metabolism of lipids and carbohydrates exhibit diurnal rhythmicity [16,43]. In addition, our data indicate that metabolic patterns are altered by chronic exposure to high-fat and high-carbohydrate diets; moreover, the magnitude of the changes depends on the stage of life at which the exposure occurred, as well as the number of exposures to an HFCD. In our study, the most noticeable effects in the adult rabbits exposed to the HFCD were phase shifts in the temporal profiles of carbohydrates and lipids, and, in some cases (such as CHOL and HDL), a loss of rhythmicity and an increase in the circulating levels of CHOL and LDL. Similar findings have been reported in mice, where the intake of high-fat diets is associated with changes in both the temporal and plasmatic levels of metabolic variables [16]. Maternal overnutrition before and during pregnancy was associated with the loss of the diurnal carbohydrate metabolism, while the lipid metabolism exhibited phase shifts in diurnal rhythmicity only, suggesting that prenatal exposure to an HFCD could be associated with alterations in the conformation and performance of the circadian system. Similar findings have been reported in rodents, where maternal nutritional conditions, either undernutrition or overnutrition, have an impact on the periodicity of metabolic variables [33,44]. Finally, the group that received the HFCD both prenatally and in adulthood exhibited a total loss of rhythmicity in both the carbohydrate and lipid metabolism, as well as an increase in their metabolic rate; thus, in addition to the loss of diurnal patterns, circulating levels of metabolites such as glucose, total cholesterol, and LDL cholesterol were upregulated, suggesting that both homeostatic and chronostatic mechanisms were compromised due to the double exposure to the HFCD.

Substantial experimental evidence shows that the ingestion of high-fat diets is associated with the development of hepatic steatosis and kidney injury [45]. Generally, these conditions are determined by measuring hepatic and/or renal damage markers at the serum level. However, traditionally, these evaluations are performed by measuring their levels at a single time of the day, without considering the fact that these markers exhibit variations throughout the day, as has been demonstrated in the case of urea rates in rabbits [46] and rodents [47], total proteins in rabbits [48], and albumin and creatinine [49]. It is possible that these alterations in the diurnal patterns of tissue damage markers are the first alterations associated with the consumption of HFCD prior to a clear alteration in the functioning of these organs. In the case of our rabbits, in the assessment of the markers of hepatic, renal, and muscular damage, there was a prevailing change in the diurnal temporality of the markers, but only CK exhibited an evident increase in its plasma levels, suggesting that muscle is more sensitive to diet. Further studies are needed to determine the prognostic value of examining the circadian patterns of tissue damage markers to determine the progression of tissue-specific diseases [50,51].

In rabbits, there are no previous studies on which we can rely to understand the absence of rhythmicity in GGT levels. However, Ihtiyar et al. [52] conducted a study on diurnal rhythmicity in different biochemical variables in humans and also reported the absence of rhythmicity in this analyte. However, in Wistar rats, the rhythmicity of GGT has been reported but only in urine [53], so it would be interesting to perform GGT determinations in rabbit urine, to establish with more accuracy the absence of rhythmicity.

With regard to the expression of core clock genes in our study, we found that maternal conditions and metabolic challenge have differential effects on both the tissues studied and the molecular elements of the circadian clock. In the case of the basal part of the anterior hypothalamus, which includes the circadian pacemaker, the SCN was the tissue in which clock gene expression had the lowest susceptibility to alterations due to maternal conditions or nutritional challenges. In different animal models, it has been widely reported that high-fat diets or food restriction have no effect on the SCN [16,38]; however, the SCN indisputably contributes to the generation and maintenance of circadian regulation at the metabolic level and the coordination of peripheral oscillators for circadian homeostasis.

The existence of circadian rhythmicity in the gut has been studied for more than a decade. In rodents, clock genes are abundantly and rhythmically expressed in all regions of the gut, such as the colon, ileum, distal jejunum, proximal jejunum, and duodenum. In addition, changes in the feeding schedule shift the phase of clock gene expression [54,55,56,57,58]. However, the administration of HFCDs seems to differentially affect the molecular machinery in the distal segments of the gut peripheral oscillator. For example, in mice, the administration of a high-fat diet to the distal ileum decreases the expression of *Cry1* and *Clock*, and, in the cecum, it only reduces the expression of *Bmal1* [59,60].

This is the first report about the susceptibility of the peripheral oscillator located in the duodenum to maternal exposure to nutritional content, since offspring obtained from dams fed an HFCD exhibited an upregulation of the *Cry1*, *Bmal1*, and *Clock* genes during the light phase. On the other hand, when the animals received both prenatal and postnatal exposure to an HFCD, the effects in the duodenum were considerably different, since they caused a downregulation of *Per1* during the dark phase. Our data suggest that the molecular machinery of the oscillator contained in the duodenum is sensitive to the nutritional content of the diet, and the impacts are dependent on the time window at which exposure to an HFCD occurs.

As in other animal models, the intake of high-fat diets during adulthood leads to significant changes in the expression of different components of the molecular machinery in WAT [16,61]. In rabbits, an HFCD in adulthood produced an increase in *Bmal1* and a decrease in *Clock*. Regarding the programming of adipose tissue by maternal conditions, our results differ from those reported based on murine models, where offspring obtained from females fed with cafeteria or high-fat diets do not exhibit changes in clock gene abundance in WAT [33,62]. In the case of the rabbits, notable changes were observed in fat, since the offspring obtained from malnourished dams exhibited an upregulation of *Cry1* and *Bmal1*, predominantly in the dark phase. These differences may be due to the different species, the type of diets used, or the well-known metabolic differences between lagomorphs and rodents. Finally, *Bmal1* was the molecular component that was consistently altered by prenatal and postnatal exposure to an HFCD, and no cumulative effect was observed for dietary exposure during both stages of development.

The other peripheral oscillator that showed major alterations in the molecular machinery was the liver, since rabbits fed an HFCD in adulthood exhibited upregulated *Per1* and *Clock* expression in the liver. There is a large body of experimental evidence demonstrating that the functioning of the liver’s oscillatory molecular clock can be modulated by the intake of Western, hypercaloric, and high-fat diets [16,63,64,65]. There is still controversy regarding the effect of exposure to high-calorie or high-fat diets during pregnancy on the expression of clock genes in the liver in murine models [33,36,37,63,64], but, in the case of rabbits, the offspring obtained from HFCD dams exhibited increased expression of *Per1* in the liver in both phases of the cycle, indicating that maternal malnourishment impacts the prenatal programming of the peripheral oscillator contained in the liver. Changes in the expression of the three clock genes under consideration were found in the livers of the group of rabbits that were obtained from mothers fed an HFCD and that were subjected to metabolic challenge with the diet in adulthood, similar to the findings about other species [36,63,64]. These changes in the liver oscillator molecular machinery may be closely related to the changes in the temporal profiles of analytes associated with the carbohydrate and lipid metabolism found in the rabbits, most of which showed the loss of 24 h rhythmicity.

## 5. Conclusions

The results presented in this article show that a maternal HFCD during pregnancy, a second exposure to a metabolic challenge with an HFCD, or both result in the long-term misalignment of diurnal rhythmic metabolic and damage markers. In addition, these changes are associated with alterations in the molecular machinery of circadian rhythmicity. Central and peripheral oscillators seem to be differentially susceptible to an HFCD, as well as to the window of time in which exposure occurs. It is possible that an HFCD has an impact on the mechanisms of generation of circadian rhythms at different levels, in such a way that this results in an uncoupling of the circadian oscillators distributed in the organism. However, there is a need for further studies to obtain the complete temporal profiles of the expression of clock genes.

## Figures and Tables

**Table 1:** Description of primers used in the RT-qPCR analysis of gene expression.

		Primer Sequence		
		Forward (5′ > 3′)	Reverse (5′ > 3′)	Probe
*Per1*	Target	CGCTACAGGCACATTCAAAGC	GGGCCACCTCCAGTTCTG	TTCCCAGCCAGTCCC
*Cry1*	Target	GTTCCCCTCCTCTTTCTCTTTATGG	CGTGGATTATTTGTTGCTGTAT	CACGCCACAATAGCTG
*Bmal1*	Target	AAGACAACGAACCAGACAACGA	CCGTTCACTGGCTGTGGAA	TCGGACGGCTGCACTC
*Clock*	Target	CACGAGAAGCCAGCACAGA	ACACACCAACAGACGGAGAATTT	TCGGCAGCCAGTCCAT

## Data Availability

The datasets generated and analyzed during the current study are included in this published article or available from the corresponding author on reasonable request.

## Supplementary Materials

The following supporting information can be downloaded at https://www.mdpi.com/article/10.3390/biology14050541/s1: Figure S1. Clock gene expression. Expression of Per1, Cry1, Bmal1, and Clock genes in the hypothalamus, liver, duodenum, and mesenteric fat of adult rabbits from dams fed a standard diet or a high-fat, high-carbohydrate diet that were metabolically challenged with SD or HFCD as the challenge diet in adulthood. Rabbits from dams fed a standard diet (SD—blue bars) or a high-fat, high-carbohydrate diet (HFCD—red bars) that were metabolically challenged with SD or HFCD (blue and red striped bars). The white stripe illustrates the light phase, and the black stripe represents the dark phase. The data were obtained in triplicate (SD/SD n = 7, SD/HFCD n = 7, HFCD/SD n = 9, and HFCD/HFCD n = 10). a denotes *p* < 0.05 vs. the SD-SD group, b denotes *p* < 0.05 vs. the SD-HFCD group, and c denotes *p* < 0.05 vs. the HFCD-SD group. Significant differences # *p* < 0.05 vs. night phase. Mean ± SEM. Table S1. Dietary nutrient composition for the standard and high-fat, high-carbohydrate diets. Table S2. Cosinor analysis of metabolic parameters obtained of F1 male rabbits at 470 days of age obtained from does fed standard (SD) or high-fat, high-carbohydrate diet (HFCD) during pregnancy, and challenged with the HFCD during 30 days. Two groups of pups from SD mothers were fed with either SD or HFCD as the challenge diet, whereas two groups of pups from mothers fed HFCD were fed with either SD or HFCD, resulting in SD/SD, SD/HFCD, HFCD/SD, and HFCD/HFCD groups. Table S3. Cosinor analysis of plasmatic levels of liver damage markers obtained from F1 male rabbits at 470 days of age obtained from does fed standard (SD) or high-fat, high-carbohydrate diet (HFCD) during pregnancy, and challenged with the HFCD during 30 days. Rabbits obtained from SD mothers fed with either SD or HFCD as the challenge diet, whereas two groups of pups from mothers fed HFCD were fed with either SD or HFCD, resulting in SD/SD, SD/HFCD, HFCD/SD, and HFCD/HFCD groups. Table S4. Cosinor analysis of plasmatic levels of kidney damage markers obtained of F1 male rabbits at 470 days of age obtained from does fed standard (SD) or high-fat, high-carbohydrate diet (HFCD) during pregnancy, and challenged with the HFCD during 30 days. Two groups of pups from SD mothers were fed with either SD or HFCD as the challenge diet, whereas two groups of pups from mothers fed HFCD were fed with either SD or HFCD, resulting in SD/SD, SD/HFCD, HFCD/SD, and HFCD/HFCD groups. Table S5. Cosinor analysis of plasmatic levels of muscle damage marker creatine kinase (CK) obtained of F1 male rabbits at 470 days of age obtained from does fed standard (SD) or a high-fat, high-carbohydrate diet (HFCD) during pregnancy, and challenged with the HFCD during 30 days. Two groups of pups from SD mothers were fed with either SD or HFCD as the challenge diet, whereas two groups of pups from mothers fed HFCD were fed with either SD or HFCD, resulting in SD/SD, SD/HFCD, HFCD/SD, and HFCD/HFCD groups.
