# Supplementary material for: Chronic Maternal Overnutrition and Nutritional Challenge in Adult Life Disrupt Metabolic Diurnal Rhythmicity and Clock Gene Expression in Central and Peripheral Circadian Oscillators
Source: Biology (Basel). 2025 May 13;14(5):541. doi: 10.3390/biology14050541 (PMC12108715; doi:10.3390/biology14050541)
Supplement: Supplementary file 1 [file biology-14-00541-s001.zip › Table S1.pdf]

Table S1. Dietary nutrient composition for the standard and high-fat and carbohydrate diets.

Composition	Standard diet (SD)	High-fat and carbohydrates diet (HFCD)
**Digestible carbohydrates (Nitrogen free extract)**	47.8%	52.6%
**Fat (Ether extract)**	3.8%	5.6%
**Crude protein**	15.7%	11.2%
**Crude fiber**	15.3%	10.2%
**Mineral (Ashes)**	11.5%	7.6%
**Humidity**	5.6%	12.8%
**M. E. kcal/g**	**2.543**	**2.610**
