# Supplementary material for: Chronic Maternal Overnutrition and Nutritional Challenge in Adult Life Disrupt Metabolic Diurnal Rhythmicity and Clock Gene Expression in Central and Peripheral Circadian Oscillators
Source: Biology (Basel). 2025 May 13;14(5):541. doi: 10.3390/biology14050541 (PMC12108715; doi:10.3390/biology14050541)
Supplement: Supplementary file 1 [file biology-14-00541-s001.zip › Table S2.pdf]

Table S2. Cosinor analysis of metabolic parameters obtained of F1 male rabbits at 470 days of age obtained from does fed standard (SD) or high-fat and carbohydrate diet (HFCD) during pregnancy, and challenged with the HFCD during 30 days. Two groups of pups from SD mothers were fed with either SD or HFCD as the challenge diet, whereas two groups of pups from mothers fed HFCD were fed with either SD or HFCD, resulting in: SD/SD, SD/HFCD, HFCD/SD and HFCD/HFCD groups.

	Group	Mesor	Acrophase (h)	% Rhythmicity	*p*	Δφ(h) vs SD/SD
**GLU**	SD/SD	137.3	09:39	91.2	<0.001*	
	SD/HFCD	146.5	15:59	73.1	0.03*	- 06h 21m
	HFCD/SD	136.9	10:14	50.6	0.1	
	HFCD/HFCD	154.1	23:29	11.5	0.3	
**CHOL**	SD/SD	47.4	18:32	98.8	<0.001*	
	SD/HFCD	103.4	22:31	34.7	0.2	
	HFCD/SD	47.3	24:52	62.3	0.07	
	HFCD/HFCD	96.4	17:17	54.7	0.1	
**LDL**	SD/SD	5.7	16:54	99.9	<0.001*	
	SD/HFCD	22.5	18:20	90.6	0.004*	- 01h 26m
	HFCD/SD	7.1	24:10	91.7	0.003*	- 07h 16m
	HFCD/HFCD	31.1	20:46	64.9	0.06	
**HDL**	SD/SD	29.6	14:05	86.8	0.008*	
	SD/HFCD	25.6	19:05	61.6	0.07	
	HFCD/SD	30.4	24:19	25.6	0.2	
	HFCD/HFCD	32.7	19:27	55.3	0.09	
**FFA**	SD/SD	31.2	08:03	82.6	0.01*	
	SD/HFCD	28.9	08:43	95.6	<0.001*	- 40m
	HFCD/SD	33.4	23:30	60.2	0.07	
	HFCD/HFCD	28.6	05:19	99.9	<0.001*	+ 03h 44m

**TAG**	SD/SD	62.3	13:41	94.6	0.001*	
	SD/HFCD	49.9	23:38	66.4	0.05*	- 09h 57m
	HFCD/SD	58.7	02:43	65.4	0.05*	+ 10h 58m
	HFCD/HFCD	56.3	18:43	37.7	0.2	

Glucose (GLU), cholesterol (CHOL), low-density lipoproteins (LDL), high-density lipoproteins (HDL), free fatty acids (FFA), and triglycerides (TAG).
p = probability
$\Delta\varphi$(h)= phase shift in hours
