# Supplementary material for: Chronic Maternal Overnutrition and Nutritional Challenge in Adult Life Disrupt Metabolic Diurnal Rhythmicity and Clock Gene Expression in Central and Peripheral Circadian Oscillators
Source: Biology (Basel). 2025 May 13;14(5):541. doi: 10.3390/biology14050541 (PMC12108715; doi:10.3390/biology14050541)
Supplement: Supplementary file 1 [file biology-14-00541-s001.zip › Table S3.pdf]

Table S3. Cosinor analysis of plasmatic levels of liver damage markers obtained of F1 male rabbits at 470 days of age obtained from does fed standard (SD) or high-fat and carbohydrate diet (HFCD) during pregnancy, and challenged with the HFCD during 30 days. Rabbits obtained from SD mothers fed with either SD or HFCD as the challenge diet, whereas two groups of pups from mothers fed HFCD were fed with either SD or HFCD, resulting in: SD/SD, SD/HFCD, HFCD/SD and HFCD/HFCD groups.

	Group	Mesor	Acrophase (h)	% Rhythmicity	*p*	Δφ(h) vs SD/SD
**T-BILI**	SD/SD	6.7	10:08	88.3	0.006*	
	SD/HFCD	6.9	19:52	69.5	0.04*	- 09h 44m
	HFCD/SD	8.3	09:51	99.9	<0.001*	+ 00h 17m
	HFCD/HFCD	7.1	18:58	43.5	0.16	
**AST**	SD/SD	23.4	13:52	74.8	0.03*	
	SD/HFCD	18.3	20:45	99.3	<0.001*	- 06h 53m
	HFCD/SD	27.9	02:14	59.2	0.08	
	HFCD/HFCD	14.3	17:13	50.6	0.12	
**ALT**	SD/SD	62.8	12:29	91.1	0.003*	
	SD/HFCD	41.9	5:31	79.4	0.02*	+ 06h 58m
	HFCD/SD	38.1	9:15	79.0	0.02*	+ 03h 14m
	HFCD/HFCD	32.9	10:01	87.2	0.008*	+ 02h 28m
**GGT**	SD/SD	4.1	9:00	65.8	0.06	
	SD/HFCD	5	19:43	0.0	0.5	
	HFCD/SD	3.6	10:11	70.4	0.06	
	HFCD/HFCD	5.6	22:40	5.8	0.4	

Total bilirubin (T-BILI), aspartate aminotransferase (AST), alanine aminotransferase (ALT), gamma-glutamyl transferase (GGT).
p = probability. Δφ(h)= phase shift in hours
