# Supplementary material for: Chronic Maternal Overnutrition and Nutritional Challenge in Adult Life Disrupt Metabolic Diurnal Rhythmicity and Clock Gene Expression in Central and Peripheral Circadian Oscillators
Source: Biology (Basel). 2025 May 13;14(5):541. doi: 10.3390/biology14050541 (PMC12108715; doi:10.3390/biology14050541)
Supplement: Supplementary file 1 [file biology-14-00541-s001.zip › Table S4.pdf]

Table S4. Cosinor analysis of plasmatic levels of kidney damage markers obtained of F1 male rabbits at 470 days of age obtained from does fed standard (SD) or high-fat and carbohydrate diet (HFCD) during pregnancy, and challenged with the HFCD during 30 days. Two groups of pups from SD mothers were fed with either SD or HFCD as the challenge diet, whereas two groups of pups from mothers fed HFCD were fed with either SD or HFCD, resulting in: SD/SD, SD/HFCD, HFCD/SD and HFCD/HFCD groups.

	Group	Mesor	Acrophase (h)	% Rhythmicity	*p*	Δφ(h) vs SD/SD
**U**	SD/SD	10.9	10:53	95.0	0.001*	
	SD/HFCD	9.3	06:43	83.2	0.01*	+ 04h 10m
	HFCD/SD	10.4	22:47	86.3	0.009*	- 11h 54m
	HFCD/HFCD	8.1	01:35	97.3	< 0.001*	- 13h 32m
**CREA**	SD/SD	150	17:38	93.0	0.002*	
	SD/HFCD	140.3	01:57	99.0	< 0.001*	- 08h 21m
	HFCD/SD	137.5	09:16	83.0	0.01*	+ 08h 22m
	HFCD/HFCD	137.8	21:36	81.1	0.01*	- 03h 58m
**T PROT**	SD/SD	62.0	12:16	99.7	< 0.001*	
	SD/HFCD	66.2	18:53	94.4	0.001*	- 06h 37m
	HFCD/SD	62.1	05:38	97.1	0.0004*	- 17h 22m
	HFCD/HFCD	55	05:59	NA	NA	
**A**	SD/SD	43.2	12:05	87.1	0.008*	
	SD/HFCD	56.5	09:49	19.5	0.3	
	HFCD/SD	47.6	05:26	99.9	< 0.001*	- 17h 21m
	HFCD/HFCD	45.8	04:29	99.9	< 0.001*	- 16h 24m

Urea (U), creatinine (CREA), total protein (T PROT), albumine (A).
p = probability
Δφ(h)= phase shift in hours
