# Supplementary material for: Chronic Maternal Overnutrition and Nutritional Challenge in Adult Life Disrupt Metabolic Diurnal Rhythmicity and Clock Gene Expression in Central and Peripheral Circadian Oscillators
Source: Biology (Basel). 2025 May 13;14(5):541. doi: 10.3390/biology14050541 (PMC12108715; doi:10.3390/biology14050541)
Supplement: Supplementary file 1 [file biology-14-00541-s001.zip › Table S5.pdf]

Table S5. Cosinor analysis of plasmatic levels of muscle damage marker creatine kinase (CK) obtained of F1 male rabbits at 470 days of age obtained from does fed standard (SD) or a high-fat and carbohydrate diet (HFCD) during pregnancy, and challenged with the HFCD during 30 days. Two groups of pups from SD mothers were fed with either SD or HFCD as the challenge diet, whereas two groups of pups from mothers fed HFCD were fed with either SD or HFCD, resulting in: SD/SD, SD/HFCD, HFCD/SD and HFCD/HFCD groups.

	**Group**	**Mesor**	**Acrophase (h)**	**% Rhythmicity**	***p***	**Δφ(h) vs SD/SD**
**CK**	SD/SD	110.6	01:17	97.1	< 0.001*	
	SD/HFCD	472	01:59	88.1	0.007*	- 00h 42m
	HFCD/SD	659.2	00:44	81.7	0.01*	+ 00h 33m
	HFCD/HFCD	382.6	04:49	99.8	< 0.001*	- 03h 32m

p = probability
Δφ(h)= phase shift in hours
